# Montreal

**Published:** 1899-06

**Authors:** James McGregor

**Affiliations:** Secretary-Treasurer


### MONTREAL VETERINARY MEDICAL ASSOCIATION.

THE regular meeting was held in the Library of the College. The President, Dr. Adami, occupied the chair. There were also present the Hon. President, Dr. D. McEachran, and Drs. Baker and Sugden.

After reading of the minutes of last meeting and disposing of other routine business, the chairman called upon Dr. Sugden for his case report on "Intestinal Obstruction." Subject was a bitch (collie), about seven months old. This dog had been taken suddenly and noticeably ill while playing in the street, uttering most heart-rending cries and showing signs of great pain. She was given ½ gr. of morphine and brought to the college. Enemas were used freely and a dose of castor oil administered. Temperature, 107.° As the pain had not moderated in the slightest degree, another ¼ gr. of morphine was injected, and this seemed to have no effect, so twenty minutes later another ½ gr. was given. All the time the dog was moaning and frequently yelling with pain, while pressure over the region of the stomach increased the pain, and produced violent straining. Feeling that a foreign body was the cause of all this pain, she was given another dose of castor-oil, combined with 30 drops of tincture of opium. The dog had now been in violent pain for two hours, and beginning to feel somewhat desperate, he gave her another ½ gr. of morphine, but this had no effect, and she was then put under the influence of the A. C. E. mixture, and kept her unconscious for about seventy minutes, by which time the morphine had produced the desired effect. This was about 10.30 P.M., the dog having had the first dose about 8 o'clock. At half-past one she was sleeping, only remitting an occasional groan. The next morning, to his surprise, he found her alive and in a semi-conscious state, but apparently free from pain. At 8 o'clock she received a little beef-tea, and shortly afterward her bowels moved slightly, and by evening she was sufficiently recovered to walk around, and her owner took her home, as he did not want to go to any further expense. Later Dr. Sugden heard that on the morning following her removal from the college she passed a potato as big as a pigeon's egg, and a worm which the owner described as being six to eight inches long, with a green back and white belly, but unfortunately both potoato and worm had been thrown away. Had twice seen the patient since her removal, and she was enjoying the best of health. This was followed by an essay by Mr. Gellatly on "Meat and Milk of Tuberculous Animals as a Menace to Public Health," and proved to be a most interesting and valuable paper. He considered tuberculosis, as compared with other diseases to which man was liable, to be the one which must be recognized as deserving the greatest attention from sanitarians, health officers, and physicians. It was found to be one of the oldest diseases mentioned, as even Moses in his law forbade the consumption of the meat of animals effected by phthisis, and at different later dates frequent mention relative to this disease was made. Tuberculosis was found to exist in every type and breed of cattle, and found its easiest victims among those kept especially for milk purposes. The homes of the wealthiest in the world and the homes of the poorest testify that our meat and milk are the cause of thousands of deaths from this disease every day. If all the victims of consumption who die annually lived in one country, newspapers would be crowded with stories about the most dreadful pestilence that ever visited the earth. Insurance companies would testify that a great proportion of the deaths in their respective orders were due to tuberculosis, and these deaths were found to be among healthy men who had been examined as to their soundness when admitted to the order. Some may have had latent tuberculosis when examined, but it can be proved that many contracted the disease after examination, and it can be traced to the eating of milk and meat of tuberculous animals. Mr. Gellatly cited instances from observations recorded by various authors who had proved that the germ of tuberculosis existed in milk. It was also shown to have lived in butter for a period of 120 days, and in cheese for as long as 35 days. A Berlin bacteriologist had been successful in inoculating a series of 250 guinea-pigs from butter purchased at random on the market-place, and pathologists have found that at least one-fifth of the consumptive diseases prevalent among children can be traced to the infection of milk. Mr. Gellatly said it had not been fully demonstrated that the disease could be contracted from eating meat of tuberculous animals, but numerous experiments had been successful in inoculating various animals with the disease from the juice pressed out of underdone steak. Heat, he said, might destroy the vitality of the bacilli if carried up to a certain point; but in many cases meat was preferred rare, and in cooking meat in joints a great part of it was not raised to a sufficient heat to destroy the bacilli. Mr. Gellatly went on to discuss the contagious nature of the disease and the various ways it could be transmitted, but contended that there was no doubt that the largest part of the tuberculosis which man obtains through his food is by means of milk containing tuberculous matter. As to the remedy, the essayist said he could not suggest anything further than that laid down on pages 7, 8, and 11 of a little pamphet published by Dr. D. McEachran, entitled *Tuberculosis in Cattle.* In conclusion he said that the veterinary surgeon, when called upon to give his opinion, should consider the sacredness of his position, and even if he chance to offend his client, to do his duty toward his fellow man.

The chairman complimented the essayist on the preparation and delivery of his paper, and a discussion ensued, strengthened by valuable information from the President and Dr. D. McEachran from observations and experiments conducted by themselves. Dr. McEachran said his attention had been called to this disease about thirty years ago, when he found that the disease existed among cattle on the farms in the neighborhood of Montreal. At that time he read a paper on the subject before the Medico-Chirurgical Society, but very few at that time recognized the disease as communicable from animal to man. A few years ago he again read a paper before the same society on the subject, and found not a single dissenting voice from any of his remarks, and the true nature of the disease was better understood and was looked upon as very dangerous and readily communicable from animal to man.

Dr. Adami said it was not necessary to have lesions in the udder in order to have bacilli in the milk. In experiments conducted by Dr. McEachran and himself, in the case of seven out of ten cows affected by the disease, and without lesions in the udder, they found the milk virulent, and on inoculation produced the disease; that we could have the disease acquired *in utero*, as Prof. Bang had found lesions in the livers of newly-born calves.

Mr. Groves was appointed essayist for the next meeting, and Mr. Hammond to report a case.

There being no further business the meeting closed.

JAMES McGREGOR,
Secretary-Treasurer.

